# Exploring How Virtual Reality Could Be Used to Treat Eating Disorders: Qualitative Study of People With Eating Disorders and Clinicians Who Treat Them

**DOI:** 10.2196/47382

**Published:** 2024-05-14

**Authors:** Helen Bould, Mari-Rose Kennedy, Ian Penton-Voak, Lisa May Thomas, Jon Bird, Lucy Biddle

**Affiliations:** 1 Centre for Academic Mental Health Population Health Sciences, Bristol Medical School University of Bristol Bristol United Kingdom; 2 Medical Research Council Integrative Epidemiology Unit University of Bristol Bristol United Kingdom; 3 NIHR Biomedical Research Centre University Hospitals Bristol and Weston NHS Foundation Trust University of Bristol Bristol United Kingdom; 4 Gloucestershire Health and Care NHS Foundation Trust Gloucester United Kingdom; 5 NIHR Applied Research Collaboration West at University Hospitals Bristol and Weston NHS Foundation Trust Bristol United Kingdom; 6 School of Psychological Science University of Bristol Bristol United Kingdom; 7 School for Policy Studies Centre for Sociodigital Futures University of Bristol Bristol United Kingdom; 8 Pervasive Media Studio Bristol United Kingdom; 9 Department of Computer Science University of Bristol Bristol United Kingdom

**Keywords:** eating disorders, virtual reality, anorexia nervosa, bulimia nervosa, EDNOS, treatment, immersive, clinicians, qualitative data, psychoeducation, therapeutic, limitations

## Abstract

**Background:**

Immersive virtual reality (VR) interventions are being developed and trialed for use in the treatment of eating disorders. However, little work has explored the opinions of people with eating disorders, or the clinicians who treat them, on the possible use of VR in this context.

**Objective:**

This study aims to use qualitative methodology to explore the views of people with eating disorders, and clinicians who treat them, on the possible use of VR in the treatment of eating disorders.

**Methods:**

We conducted a series of focus groups and interviews with people with lived experience of eating disorders and clinicians on their views about VR and how it could potentially be used in the treatment of eating disorders. People with lived experience of eating disorders were recruited between October and December 2020, with focus groups held online between November 2020 and February 2021; clinicians were recruited in September 2021 and interviewed between September and October 2021. We took a thematic approach to analyzing the resulting qualitative data.

**Results:**

We conducted 3 focus groups with 10 individuals with a current or previous eating disorder, 2 focus groups with 4 participants, and 1 with 2 participants. We held individual interviews with 4 clinicians experienced in treating people with eating disorders. Clinicians were all interviewed one-to-one because of difficulties in scheduling mutually convenient groups. We describe themes around representing the body in VR, potential therapeutic uses for VR, the strengths and limitations of VR in this context, and the practicalities of delivering VR therapy. Suggested therapeutic uses were to practice challenging situations around food-related and weight/appearance-related scenarios and interactions, to retrain attention, the representation of the body, to represent the eating disorder, for psychoeducation, and to enable therapeutic conversations with oneself. There was a substantial agreement between the groups on these themes.

**Conclusions:**

People with lived experience of eating disorders and clinicians with experience in treating eating disorders generated many ideas as to how VR could be used as a part of eating disorders treatment. They were also aware of potential limitations and expressed the need for caution around how bodies are represented in a VR setting.

## Introduction

Eating disorders are serious mental illnesses, affecting around 8% of women and 2% of men at some point in their lives [1]. They have substantial physical [2,3] and psychiatric [4] comorbidities, with anorexia nervosa having the highest mortality of any psychiatric illness [5]. Nationally representative population data from England show that rates of possible eating disorders in adolescents almost doubled from 6.7% in 2017 to 13.0% in 2021 [6]. Meanwhile, at 10-year follow-ups, only one-third of those with anorexia nervosa and two-thirds of those with bulimia nervosa have fully recovered [7], and rates of relapse are up to 50% in those with anorexia nervosa [8]. New treatments are thus urgently needed.

Virtual reality (VR), a technology which “immerses users in a fully digital environment through a headset or surrounding display” (p. 3) [9] is a candidate modality for such novel treatments. VR shows promise in treating mental illnesses, with studies demonstrating efficacy in treating phobias, including fear of heights [10] and spiders [11]; anxiety disorders [12]; and avoidance and distress in people with psychosis [13]. Emerging evidence suggests that VR can be useful in the treatment of eating disorders, with a meta-analysis showing reductions in binge frequency and body dissatisfaction in binge-purge and binge-eating disorders [14]. Some evidence also suggests that exposure to a larger version of one’s own body in VR may be a useful addition to the treatment of anorexia nervosa [15,16].

One problem with the currently established treatments for eating disorders is the high rate of dropouts. Even in randomized controlled trials, as many as 40% of participants do not complete treatment for anorexia nervosa [17]. For new treatments to have the best chance of being acceptable to patients, a prerequisite for efficacy, they should be designed in collaboration with the patient group for whom they are intended. To our knowledge, no previous work has explored the perspectives of people with lived experience of eating disorders on the potential use of VR in their treatment.

There is also a longstanding acknowledgment of the difficulty in ensuring that patients receive evidence-based treatments, variously termed an “evidence to practice gap” [18], “implementation problem” [19], or “second translational gap” [20]. This is a particular challenge for complex interventions. To avoid or shorten this gap, it is argued that new interventions should be designed with implementation in mind and that frontline clinicians should also be involved in novel intervention design from the outset. In relation to the growing field of developing clinical interventions using VR, an international working group of Virtual Reality Clinical Outcomes Research Experts also state that “it is vital to include the patients’ voice early and often in the development of VR treatments” (p. 2), as well as seeking input from health care providers, to design acceptable, feasible, and effective VR treatments [21].

We therefore set out to conduct a series of focus groups and one-to-one interviews with both people with lived experience of eating disorders and, separately, the clinicians who treat them. We explored their thoughts about using a VR environment, including their ideas about the potential therapeutic uses of VR, and any concerns or worries about risks.

## Methods

### Participants

We recruited participants with lived experience of eating disorders via social media, through the UK eating disorders charity the Somerset and Wessex Eating Disorder Association, and by contacting those on a list of research-interested individuals with eating disorders held by HB. We recruited clinicians with experience in treating people with eating disorders via social media and by snowballing via professional contacts. Potentially interested participants completed a brief online screening questionnaire via SurveyMonkey (SurveyMonkey Inc.; Multimedia Appendices 1 and 2) to check they met inclusion criteria.

Inclusion criteria for people with lived experience of an eating disorder required that they be aged 16 or over, and have any current or previous eating disorder (eg, anorexia nervosa, bulimia nervosa, binge eating disorder, or other specified feeding or eating disorder). We did not specify that participants must be based in the United Kingdom. We excluded people currently being treated as an inpatient in a hospital. Inclusion criteria for clinicians required that they be a health professional with at least six months’ experience in treating people with eating disorders. Inclusion criteria for both groups required fluency in English (due to a lack of funding for translators) and the ability to access a private space with an internet-enabled device via which they could join an online focus group or interview. We did not require participants to have any previous experience of using VR. We conducted interviews and focus groups online as a result of constraints around in-person research during the COVID-19 pandemic. All participants provided written consent and were offered a £10 (US $12.5) shopping voucher to thank them for their time.

After recruiting the first 8 eligible participants who were women and who responded to follow-up emails, we excluded subsequent responders who were women to include men. All eligible clinicians who responded to follow-up emails were included.

People with lived experience of eating disorders were recruited between October and December 2020, and focus groups were held online between November 2020 and February 2021; clinicians were recruited in September 2021 and interviewed between September and October 2021.

### Ethics Approval

The study was approved by the Faculty of Health Science Research Ethics Committee, University of Bristol (reference number 7545).

### Procedure

We collected brief demographic data, including age, gender (participants were asked “How would you describe your gender?”) and ethnicity, duration of current or previous eating disorder (for people with lived experience of eating disorders), professional background (for clinicians), and duration of experience in treating people with eating disorders (clinicians).

Interview and focus group discussion topics were informed by a topic guide (available in Multimedia Appendices 3 and 4). We also shared some slides with images of VR headsets and from some VR games and current VR interventions for those with fear of heights and psychosis (see Multimedia Appendix 5). We used open-ended questioning and follow-up probes to explore participants’ ideas in detail and also allowed them to suggest and discuss issues of importance that were not included in the topic guide. All interviews and focus groups were then conducted via videoconferencing software (Microsoft Teams; Microsoft Corporation), audio-recorded on an encrypted recording device, and transcribed verbatim. Authors HB and LB ran the focus groups together, enabling one of them to monitor the written “chat” function and provide support to any distressed participant if necessary. HB conducted the one-to-one interviews alone.

### Analysis

We used a thematic approach, with MRK, HB, and LB all taking part in coding. These coauthors brought differing perspectives and expertise to the data: LB as an associate professor in qualitative mental health research, MRK as a more junior qualitative researcher in health and ethics, and HB as a child and adolescent psychiatrist with research and clinical expertise in eating disorders. HB’s clinical expertise likely influenced the lines of inquiry taken within the interviews and allowed greater probing of the participants, but may have introduced bias. This clinical perspective is likely to have been counterbalanced by LB and MRK approaching the interviews and analyses from their complementary and nonclinical perspectives. MRK conducted the initial coding of 2 focus groups. Sections of the data from these 2 focus groups were then coded separately by coauthors LB and HB, with high levels of concordance in the coding. LB, HB, and MRK held a series of meetings to finalize the coding frame, and MRK then applied this frame to the final focus group. As the coding frame evolved, codes were grouped to derive themes and subthemes.

MRK then applied the same coding frame to the clinician interviews. Subsections of these interviews were coded by LB and HB, and further meetings were held between HB, MRK, and LB to finalize agreement on these codes. The coding frame evolved through the addition of new ideas from clinician participants and through discussion, we grouped the codes into themes and subthemes. MRK then wrote a descriptive account of the themes and subthemes to explore in detail their content and relationships.

## Results

### Participant Characteristics

A total of 14 participants contributed to these data: 10 people with lived experience of eating disorders and 4 clinicians (Table 1). People with lived experience of eating disorders included both people with a current eating disorder and people who have recovered. The length of illness varied from 3 to 20 years. Clinicians came from a range of professional backgrounds and had between 2 and 15 years’ experience of working with people with lived experience of eating disorders. We recruited men and women in both groups.

The screening survey was completed by 31 respondents with lived experience: 5 did not fully complete the survey or supply contact details, 8 did not respond to email invitations to join a focus group, and 8 were women who responded after we had held 2 focus groups with women and were purposively sampling participants who were men. These 21 respondents were therefore not included, and the remaining 10 joined a focus group. Nine clinicians completed the screening questionnaire: 3 did not complete it fully or supply contact details, and 2 did not respond to email invitations.

We held a total of 3 focus groups for people with lived experience of eating disorders: 2 with 4 participants and 1 with 2 participants. As a result of difficulties in finding mutually convenient times, we were not able to hold clinician focus groups and instead held a series of one-to-one interviews.

**Table 1:** Participant characteristics.

Characteristics			Values
**People with lived experience of eating disorders (n=10)**			
	**Gender**		
		Women, n	8
		Men, n	2
	Age (years), range		19-37
	**Ethnicity**		
		White/White British, n	10
	**Current eating disorders**		
		Current anorexia nervosa, n	4
		Current bulimia nervosa, n	2
		Duration (years), range	3-20
	**Previous eating disorders**		
		Previous anorexia nervosa, n	5
		Previous eating disorder not otherwise specified, n	1
		Duration (years), range	5-16
	**Recovered from eating disorders, n**		4
		Duration (years), range	0.25-6
**Clinicians (n=4)**			
	**Gender**		
		Women, n	3
		Men, n	1
	**Age (years)**		
		25-34, n	1
		35-44, n	1
		45-54, n	1
		55-64, n	1
	**Ethnicity**		
		White/White British, n	4
	Duration (years) of clinical experience with people with eating disorders, range		2-15
	**Professional background**		
		Clinical psychologist, n	1
		Counselor, n	1
		Family therapist, n	1
		Mental health nurse, n	1

### Themes

#### Overview

We describe the core themes: Representing the Self, Strengths and Limitations of VR, Potential VR Interventions, and Practicalities of Delivering VR Therapy. Quotes from people with lived experience of an eating disorder are followed by PWLE, and the number indicates their group (members of groups 1 and 2 were all women; and members of group 3 were men); quotes from clinicians are indicated CL. Most participants reported having tried VR as entertainment, and those who had not reported that they were willing to try it.

#### Representing the Self

Everyone acknowledged that deciding how best to represent the self in a VR setting was challenging (“one of the really tough questions” [PWLE3]); “need[s] to be very carefully...done” [CL4]).

People with lived experience of eating disorders felt comfortable with a first-person perspective: “I quite like the idea of just it being from my perspective, so just seeing your feet and hands” [PWLE1]; “I was imagining that you wouldn’t see yourself...you’d see what you would normally see if you were walking around” [PWLE2]. Clinicians agreed: “if I had an eating disorder, I’d probably prefer not having my body [in the VR setting], just having my hands” [CL1], also suggesting that this would increase a sense of embodiment: “[if] I just see my hands, that feels more real because when I’m me, I can’t see my body, unless I look down my body” [CL1]. Some people with lived experience of eating disorders highlighted the importance of accuracy and realism to enable them to suspend disbelief, including representing correct skin color and gender even if only representing a hand.

Some people with lived experience of eating disorders were concerned about the possibility of their whole body being represented, for example, from a third-person perspective or in a mirror, which they felt may exacerbate self-criticism and create risk: “maybe I’ll see something on VR that I’ve never seen in real life and it makes me worse...you might go, wow, I really do look shit in that outfit or you know nude or whatever” [PWLE2]; “with eating disorders being the beast they are, anything that would maybe feed into that kind of inner critic, or that sort of obsession with our sort of external appearance, it would have to be handled very sensitively...for it not to risk causing more harm than good” [PWLE1]. They were also concerned about how accurate images might be created: “wouldn’t you have to take full body photos of people...which in itself I think could be quite difficult” [PWLE1].

There was some concern that an accurately represented body might be distracting: “you would then become too focussed on what it looked like, and then ignore the job in hand” [PWLE2], but participants in both groups also discussed that this itself could form part of treatment (see the “Potential VR Interventions” section).

People with lived experience of eating disorders discussed alternative suggestions as to how their bodies may be represented, such as a “cartoon”-type avatar [PWLE2] or animal [PWLE1]. While such alternatives might be useful to avoid “getting hung up” [PWLE1] on one’s image, they were largely dismissed as unrealistic or silly, and possibly preventing engagement: “I think seeing me like a kind of panda pinballing around...I just wouldn’t...feel like I could get on board with it” [PWLE1].

Representing the self in an abstract form was also suggested and again felt to have therapeutic potential (see the “Potential VR Interventions” section): “maybe they could be shapes, maybe they could be manifested in different things, like personifications, emotions...I actually think that could be part of the process” [PWLE3].

Despite prompting, clinicians expressed few views on how representation should be achieved, tending to share the belief that “it’s best to go with their [PWLE] visualisation, because then it has more meaning” [CL1]. However, participants in both groups agreed the actual process of creating a representation of self could be challenging if this presented patients with excessive choice: “I would be distracted by worrying what my virtual [cartoon avatar] self looked like if it was down to me [to make it] or whether it was down to someone else to generate me then I might get offended” [PWLE1]. One clinician suggested this process could be simplified: “so that we didn’t have endless conversations about choice...there’s a bit of me that would go for a menu of what somebody looks like so they could choose a torso or...a torso or legs or a face...or the menu would just be certain kinds of figures” [CL3]. One clinician also raised the point that it may be important to discuss the process of leaving VR to return to one’s own real body: “we’re not going to be that avatar so, how do we then come back out into the real world and reconnect with who we really are, even the bits we don’t like” [CL1].

#### Strengths of VR

People with lived experience of eating disorders and clinicians were excited about the potential of using VR in treating eating disorders and felt it may be more engaging than other treatments: “I would be more motivated to do that as my homework than my mood diaries or my thought diaries” [PWLE3]; “I’d be really interested in it, definitely like give it a go, I imagine young people would just engage very well in it” [CL4].

Participants described the strength of creating realistic situations, which nevertheless were not actually real and so could provide a safe space to practice: “knowing it’s not real, but also it’s simulating something that is real so it would be a very good first approach to...expose somebody to something scary” [PWLE1]; “a gentle form of exposure work” [CL3]. One clinician [CL1] wondered if this might be particularly valuable for some groups of patients, suggesting that those who are autistic or anxious may find it easier to engage in a “real” activity than a face-to-face conversation.

Participants noted that in some ways VR was “better” than real life because it is a “controlled environment” [PWLE2 and CL3] and having control—specifically the ability to stop—allows users to explore or confront activities they would not feel able to try in the real world: “you can always turn it off...you’re not going to be halfway round a roller coaster having a panic attack” [PWLE2]; “you can take it off any time” [PWLE3].

People with lived experience of eating disorders suggested VR could also help make therapy more closely related to real life, by making it more engaging and action-based “I find sitting on the couch in the room [in therapy] really tough, I think it’s [VR] something physical to do, a practical thing to do” [PWLE3]. They also suggested that VR might enable therapeutic work to feel more directly connected to real life because it could enable “the therapist [to be] there with you as you’re experiencing those things...rather than...have to remember these situations and then feed them back...that’s a challenge to remember what you’ve actually thought and felt at that moment in time and then be able to share it with a therapist when you’ve got that meeting...three days later” [PWLE2]. Another person with lived experience of eating disorders explained “I’m going to benefit from maybe being in a room with my therapist and having a sort of virtual reality...challenge and her sort of...being able to be there, in the moment...I think it would help me feel like we’ve had a better understanding of what it actually feels like in that moment” [PWLE1].

Clinicians discussed the possibility of using VR alongside existing treatments “whatever that end goal is I think that it requires picking up on tools that you think are going to work for that particular individual and I just see VR as being a tool”, adding “if it was available you might be kind of picking it off the shelf” [CL3]. Relatedly, they mentioned its potential as a scalable resource, enabling more support than might otherwise be possible in overstretched services: “potentially then, we know that people are getting something additional to what they are currently able to access often in services” [CL2].

#### Limitations of VR

Some people thought VR headsets might be heavy, uncomfortable, or hard to take off quickly. Some without personal experience of VR were worried about feeling claustrophobic or “panicky” [PWLE2], though this was countered by the experience of others.

Practical concerns about being unable to see one’s surroundings and feeling off-balance were also raised, contributing to fears about feeling unsafe and “vulnerable” [PWLE1 and PWLE2] while using VR; it was felt that individuals’ experiences may increase such feelings. Participants also described potentially feeling self-conscious, a common experience when trying VR in other contexts: “I think you feel like a bit of a dick when you try it on” [PWLE3].

Both people with lived experience of eating disorders and clinicians were concerned that clinicians might not feel confident about using VR technology, and one person with lived experience of eating disorders also suggested VR was “not for everyone”: “I wouldn’t naturally be very interested in VR to be honest and I think it’s maybe for people who are younger than me” [PWLE3].

Participants also talked about technical limitations relating to the suitability of VR in treating eating disorders. Concerns included the quality of graphics, and that VR may be limited to visual and sound worlds, which would not be able to capture the physical sensations involved in experiencing and recovering from an eating disorder: “when I went through recovery...it was physically existing in a bigger body...like the physical sensation of inhabiting a bigger body...I’m not sure that VR could address” [PWLE1]; “it’s kind of bingeing and then not eating anything, it’s that sense of hunger, like you can’t replicate that with VR” [PWLE1]; “for me there’s an additionality of thinking about smells or sounds” [CL3].

Some clinicians discussed the idea that VR not being real might be unhelpful because it could facilitate avoidance of real life: “fuelling more the idea that we can be this virtual reality person that’s not ourself, because we don’t like ourself” [CL1], or avoidance of treatment: “I think with an eating disorder there’s always this kind of temptation to step away, isn’t there? And actually there is a real need to just get on in there and do it [the treatment] right away” [CL2]. However, clinicians also described this viewpoint as in “tension” with a recognition of the idea that VR could be used as an intermediate step or “tool” for people who are not yet ready to try things out in real life: “[VR] feels more accessible and then, they feel more kind of empowered and strengthened taking it forwards in the real situations” [CL2].

People with lived experience of eating disorders raised the concern that the fact that it is not real might make VR easier to dismiss: “I might be inclined to think, well that was just in VR, in real life it would be totally different” [PWLE1]; “I think it must be really difficult to...apply it because you think well that wasn’t real” [PWLE2].

One person with lived experience of eating disorders also raised the idea of VR being a fad and the possibility that it could be an expensive distraction from the need to train more therapists

#### Potential VR Interventions

Participants made a wide range of suggestions for potential VR interventions to help and treat people with eating disorders. They agreed treatments would need to vary for each individual: “ultimately it has to have meaning to the person you’re working with” [CL1]; “no pun intended, one size definitely won’t fit all” [PWLE1]. However, suggestions could be grouped according to the type of intervention.

##### Practicing Everyday Challenges

One common type of intervention discussed by participants was the idea of using VR to “sort of put yourself in that [challenging] situation as an in-between step before you end up doing it” [PWLE2]; or “practicing helpful behaviours or responses to situations...having an opportunity to try out responding in a different way...so...it could feel much easier to access that in the real world” [CL2]. Challenges fell into 3 main groups: food-related scenarios, weight/appearance-related scenarios, and challenging interactions. Examples are presented in Table 2.

Both groups discussed how VR could allow people with eating disorders to practice such challenges in a protected way, to decrease anxiety or “desensitise” [PWLE2] oneself, thus providing an in-between step before going into a real situation “there’s a whole lot of...step-based approaches to end up with sitting in a café having a piece of cake...[in VR] you’re taking away a whole layer of somebody being concerned about the public’s reaction to them” [CL3]. VR might fill an important gap between talking about doing something and actually doing it: “it’s kind of that buffer, that bridge to being exposed to those things in a real world” [PWLE1]; “it could probably lend itself quite well to practising going up to someone and saying, you know, can I have this drink or whatever, like it would be a good sort of in between step, because otherwise...there’s lots of talking and planning around it but there’s nothing in between that can, you know, reduce their anxiety” [CL4].

**Table 2:** Quotes about challenges and potential VR interventions.

Challenges and specific scenarios		Quotes describing challenges	Quotes relating to the proposed VR intervention
**Food-related scenarios**			
	Cafés	going into Costa or whatever and ordering that hot chocolate...those other challenges that are almost kind of like steps before the eating [PWLE2] having to talk to a waiter or...interact in a normal way whilst your brain is feeling anything but normal [PWLE1]	getting used to...where all the cakes are on the counter and not feeling too overwhelmed by that [PWLE2] I will never meet the person who’s in this situation as a waiter, therefore I can try saying different stuff and then you can kind of repeat the same situation [PWLE1]
	Supermarkets	I really struggled with going into a supermarket to pick a sandwich off the shelf...I get paralysed with the indecision and the kind of temptation to look at calorie labels and the knowledge I shouldn’t be [PWLE1] I would struggle with not impulsively buying loads of food for binge eating [PWLE3]	making sure like you go down each aisle...when there’s like the chocolate aisle or whatever and physically taking one off the shelf, putting it in your basket...trying not to check the calories or seeing other people’s responses, actually no one is shouting at you, oh look at the girl, she’s got chocolate in her basket...just to get exposure to those sorts of things [PWLE2]
	Eating in public	eating in front of others...if I’m in work I find that almost impossible because you think everybody’s watching you [PWLE2]	the idea of eating a real meal but in an environment created by the VR. With other people around and again that idea of are people going to be watching me...experiencing that a few times will desensitise me to go out and do it [PWLE2]
	Portion sizing	I don’t have a good sense of how much I should be eating or shouldn’t be eating [PWLE1]	serve yourself a meal from this thing and then you could then say, okay, the nutrients you’re getting from that [PWLE1] you could have a go at dishing yourself up a plate of food...and all of a sudden pull out of the cupboard what somebody might consider to be a normal plate of food and have a conversation [CL3]
**Weight/appearance-related scenarios**			
	Being weighed	I’m happy to get on my own scales now...whereas when I was anorexic it was terrible [PWLE3]	having a conversation about how do you feel...looking at those scales being now in your virtual room [PWLE3] perhaps you have an expectation or desire of what you want the number [on the scales] to be and it’s not that, or in one scenario it’s more than what you expect it to be and how would you feel about that? [PWLE3]
	Changing rooms/gyms	The environment that I felt was really difficult would be changing rooms...I just feel very self-conscious [PWLE3]	what is it like to go into a gym where everybody is extremely muscular and like how do I feel about that? [PWLE3]
	Public places	I couldn’t walk on the main roads because I thought I was too ugly...I thought...cars...would crash because I was so like shockingly hideous [PWLE3]	you could use VR to kind of emulate that feeling of being in a body and being looked at...the feeling of being observed and how to get through that [PWLE1]
	Clothes shopping	one of the biggest limitations if you go clothes shopping and obviously there’s great big mirrors everywhere... [PWLE2]	with the eating disorder maybe people can wear certain types of clothes or like baggier clothes and actually even trying on like different styles and realising what you might like or what you might like to experiment...kind of finding your own identity slightly more [PWLE2]
**Interactions**			
	Unhelpful comments	I find it really hard to know how to respond...where people are talking about say their own weight or their own eating...or...commenting on my weight or my eating [PWLE1] when they go back to school...what they’re going to say [about why they’ve been away]...we tend not to discharge anyone until they’ve got a plan around that because that feels so difficult for them [CL4]	having your therapist there with you going look, okay, so they’ve said that that’s made me think that what, yeah, how do I work this through [PWLE1]
	Asking for help	for me, asking for help was really, really, hard [PWLE3]	if we had a virtual reality scenario where I go and they don’t give a great response...I can practise how I’m going to respond to that...it could be about easy steps, like level one, you ask for help and somebody does like whatever...and then other situations where it’s more difficult...or more confrontational [PWLE3]
	Practice skills	—^a^	a virtual environment to practise...skills that they have in DBT [dialectical behaviour therapy] would have been great [PWLE3] practice anything in terms of like life skills...I think mostly like communicating within a family...practising for what you want [CL4]

^a^Not available.

Both groups also suggested that VR could be used to try out these situations at different levels of difficulty. For example, other avatars in a VR setting could be used to make challenges easier: “maybe you can have someone in the queue before you ordering that and kind of normalising that” [PWLE2], and situations could also be made increasingly challenging: “graded exposure of challenge...depending on what the fear is, is it talking to other people, is it asking for food, is it having the food in front of you. Like any of those number of things you could recreate in virtual reality” [CL4].

VR could also enable discussion about in-the-moment thoughts and feelings about being in a feared situation, in a way that would be more immediate than talking retrospectively in a therapy session: “going as a VR to a restaurant and then you’re talking about, how is it making you feel, the fact the person is eating...might be a useful tool” [CL1]; “you could look at a menu and in the session you can kind of go, okay, what’s going through your head and you do it as a CBT [cognitive behavioural therapy] thing” [PWLE1]; “being able to expose yourself to kind of challenging situations like that and being able to talk through what your instant thoughts are and then I guess rationalise them a bit more could be helpful” [PWLE2].

Most people with lived experience of eating disorders and clinicians agreed that this could be useful, though a clinician raised a concern: “is it something that would mean they [patients] could practice it and then [be able to do it] or would [it contribute to feeling that] everything has to be done perfect?” [CL4].

##### Attention

Several people with lived experience of eating disorders described how their attention was affected by their eating disorder, causing them to fixate when in public on, for example, people with particular body types, or food outlets: “it feels very bizarre to me now but like, I remember walking down the high street and it was like I had a zoom cam in my head, like spot the skinniest person in the street, and then I would focus on them all the time and then compare myself to them” [PWLE1].

They suggested VR could be used to help individuals recognize what they were attending to: “I don’t know if you can like eyetrack people over the VR” [PWLE2]; “it would be a really useful tool to kind of just confirm that bias” [PWLE2]. Building on this, they suggested VR might then be used to retrain attention: “actually if they’re saying...we’ve just noticed you’re looking at this, how about looking at those” [PWLE2]; “it could be a good way of getting rid of those biases and try to see things in a more kind of healthy and realistic way” [PWLE2].

##### Representing the Body

Body representation was discussed in terms of whether it could be a therapeutic intervention. The suggestions, why they were felt to be potentially helpful, and supporting quotes are presented in Table 3. The ideas comprised representing the body in an abstract way, having a third-person perspective of one’s body, or a third-person perspective of one’s body at different weights, and comparing self-generated versions of own body with those generated by the clinician. The area was agreed to be complex, with even the act of choosing how one’s body should be represented giving scope for therapeutic discussion around why someone wanted to be represented in a particular way: “if you allowed somebody to choose say their avatar...to get different conversations about the whys and what fors of choice...how do they think somebody would perceive the avatar and why” [CL3].

**Table 3:** Quotes relating to how representing the body could be therapeutic.

Representation of the body and how it might help		Illustrative data
**Abstract/exploratory representation of body**		
	Reduce the importance of shape and size in self-evaluation	loosen up some of the fixed ideas about your body...lessen the importance of how you view your body [PWLE3] what I looked like...was so central to maintaining anorexia that if there was something that could have helped me shake that up or reconceptualise that, that would have been great...if you could represent yourself, like maybe that’s a physical thing or maybe that’s also like I want to be courageous or I want to be strong or...I want to climb a mountain [PWLE3]
**Third-person perspective of own body**		
	Understand the severity of the illness	if I could have seen myself from the perspective of another in the past I would have been shocked...about how unwell I looked [PWLE3] I had moments [when unwell with anorexia nervosa] of like stepping out of myself and being, my goodness, I look really unwell, whereas most of the time I thought I was overweight [PWLE3]
	Change attitude to own body and reduce avoidance	practice the sorts of more positive self-talk...to override some of those really powerful negative comments that come up...but then...being able to step away from it quite easily when it feels too much...A lot of people go through life and just kind of avoid looking at their image...and actually, it’s more helpful to be able to look at yourself...focusing on the things that you do like about your body and challenging your ideas about the bits you don’t like [CL2]
**Third-person perspective of own body at different weights**		
	Reduce anxiety about planned weight restoration	seeing yourself as a bigger weight as a kind of like exposure therapy so you can kind of get your head around what that might look like. [PWLE1] ...it would be a good thing for them to see...and...explore...what they see and how they feel about that [planned weight increase] [CL4]
	Reduce anxiety around small fluctuations in weight	nowadays [in recovery]...I’d find that quite useful actually...it would be interesting to see...this is what you look like with or without like a small weight increment and you can’t tell the difference [PWLE1]
	Seeing that other people do not react as feared	you could see other people’s responses to you at that normal weight and just witness that actually people aren’t going, oh my gosh, that’s huge, or people aren’t responding at all [PWLE2]
**Compare self-generated with VR-generated image**		
	Allow comparisons and discussions	you could ask somebody...to come up with their own image about what they look like and then overlay the comparison, if somebody can tolerate that...I think those are...helpful possible conversations to have [CL3]

Various caveats were expressed. These included the idea that some interventions might not be useful for everyone: “other people might potentially find it triggering” [PWLE3], and that it may be unhelpful to focus too much on appearance “the way we look is just a small part of who we are, and there’s probably other things we want things to be doing alongside” [CL2].

Manipulating the weight of the body representation was particularly controversial. Some people with lived experience of eating disorders felt it would not be helpful, that they would not want to experience it, or that it may be counterproductive. There were concerns from both clinicians and people with lived experience of eating disorders that it might lead to increased rumination about body size, or lead to further disordered eating behaviors or weight loss: “it really could have triggered...restricting behaviours if it really kind of distressed me...kind of almost like an anxiety thing like just the fear of kind of knowing what you look like” [PWLE2]; “[if] you don’t like what you see and then you go, wow, I don’t want that and then it’s going to push you down the other way so it’s a bit of a risk” [PWLE1].

Another concern was that images of self at a healthier weight might encourage a focus on specific body parts rather than on the overall appearance of looking “well”: “they might just be focussing on, ‘Oh god, there’s no thigh gap’ you know...would they be able to see the glowing skin and the healthy hair..?” [CL2]. Some clinicians also wondered if very unwell patients would be able to make use of it, highlighting that it would need to be used in an appropriate and timely fashion: “for somebody very much in the grip of anorexia...I can imagine you’d have conversations, ‘Well, the computer’s wrong’” [CL3]. Another question was how it would be possible to know what one’s body would look like at a different weight: “you can’t really predict what that would be like just through like a computer algorithm” [PWLE1].

There was general agreement that therapeutic work around body representation would be better done with the support of a therapist: “I would only want to do it with a therapist that I trusted rather than having the option to look at it at home” [PWLE2]. This was partly because people wanted to be supported through the process, and partly because they felt it would be useful for someone else to be in control as repeatedly checking might be unhelpful “like controlling the scales and not going on the scales as often as you might otherwise.” [PWLE2].

Clinicians also felt that such an intervention would need to be used at the right time in relation to motivation and recovery: “someone that was coming in in a different mindset and...they really did want to change...then yes, maybe it would work” [CL1], and that if used it should not be a drawn out intervention but “quite time-limited or focused bit of work” [CL2].

##### Representing the Eating Disorder

One person with lived experience of eating disorders had a previous experience of using an art program in VR as a means of illustrating his eating disorder. In it, he “was trying to simulate what happens when you start eating again after starvation...it was also quite good at communicating to other people as well” [PWLE3]. He explained “I’d lived that in difficult feelings and difficult behaviours and stuff but I hadn’t been able to communicate it in words and I think that communicating it visually and using space and colour...was really powerful and therapeutic.” Part of what had been helpful was “you have the paintbrush and you are in charge, you have agency...it met some of those core needs about agency” [PWLE3]. Another person with lived experience of eating disorders agreed “this is about creativity and doing something different in the space...I think it would really appeal to me” [PWLE3]. One clinician also suggested VR could be used to help patients separate their eating disorder from their idea of themselves, for example, by creating a representation of the eating disorder in the virtual world: “that classic anorexia externalisation process of having anorexia in the room with you...There might be a place for that as well as the young person” [CL3].

##### Psychoeducation

People with lived experience of eating disorders described their family members, friends, and clinicians finding it hard to understand what they were experiencing: “so much of the pain in the heart of having an eating disorder is people around you just not understanding” [PWLE1]. They suggested that VR training could help others to understand eating disorders better: “to train therapists or charity volunteers or GPs or family to...get that kind of level of understanding and empathy” [PWLE1]. For example, VR could be used to “show the messages, pinging and pinging and pinging with all the influencing thoughts that we get...the fact that you’re focusing on you know skinny people, calorie labels, smells...” [PWLE1].

Clinicians agreed VR could be used to “increase family members’ understanding” [CL4] and “help them step into a different position” [CL3]. One clinician suggested VR could be used as part of family-based therapy, to coach parents in skills to support their child so that “the parent can go in more likely to get it right, I guess having had a bit of practice with that beforehand” [CL2]. However, they also wondered what might be lost through the young person not witnessing their parent being coached as would be her normal practice “when the young person sees the parent being coached...sometimes that’s really helpful because they know...it’s not just coming from the parent...it’s the professionals.”

One person with lived experience of eating disorders suggested that VR could be used to make learning about eating disorders more engaging for those with the illness too: “I think it would be a really good learning module...actually I find concentrating very difficult so to make learning fun and engaging and dynamic” [PWLE3].

##### Enabling Therapeutic Conversations With Oneself

People with lived experience of eating disorders raised the possibility of VR enabling them to see themselves from an external perspective which will allow them to support themselves better. It could be a “learning opportunity for you to impart some wisdom so could you kind of visualise, like create yourself, you know, is it that kind of shit that they say...would you say this to other people, what you’re saying to yourself” [PWLE3].

### Practicalities of Delivering VR Therapy

#### Overview of Opinions

Participants highlighted practical considerations around the delivery of VR therapy, which would vary according to the precise nature of the intervention. Supportive data for this section are available as Multimedia Appendix 6.

#### Setting

Opinion was divided as to whether people would rather use VR interventions at home or in the clinic. Arguments in favor of the home environment centered around it being less stressful, especially because one would not need to worry about being observed. However, others preferred the idea of a clinic setting because they felt that a potentially difficult or distressing VR experience could intrude on the safety associated with home, and some questioned whether they would use VR if left to do so independently.

#### Therapist Presence

Many participants in both groups would want therapist involvement in or alongside any VR, with people with lived experience of eating disorders highlighting that they would need to know and trust their clinician. Others suggested that the therapist’s input could be intermittent, with VR providing additional support, for example, between or alongside other therapeutic work.

#### Timing

The best timing for a VR intervention was agreed to be important, complex, and dependent on the nature of the intervention. Some felt that people with eating disorders may not be able to make use of it if they were significantly underweight or not eating, while others suggested it might be useful for people who were very underweight as it might help to manage high levels of anxiety in relation to beginning treatment. Others felt that VR interventions could be useful at any point, and highlighted that the severity of eating disorder symptoms is not always related to weight. Several participants in both groups raised the patient’s current motivation for recovery as important, feeling that this might be the most important factor in whether people would engage with and potentially benefit from VR-based treatments.

## Discussion

### Principal Findings

Both people with lived experience of eating disorders and clinicians with experience in treating eating disorders were in general positive about the possible future use of VR in eating disorders treatment. They generated a wide range of ideas as to how VR could be used, including to practice challenging situations, retrain attention, represent the eating disorder, for psychoeducation, and to enable therapeutic conversations with oneself. They discussed the complexity of how to represent people’s bodies in a VR setting and ways in which this could be done safely and potentially therapeutically. Clinicians and people with lived experience of eating disorders independently suggested similar uses for VR, and the coding framework initially developed for the focus groups with people with lived experience of eating disorders fitted well with data obtained from clinicians.

To our knowledge, this is the first study to explore the views of people with lived experience of eating disorders and clinicians about how VR could be used in the treatment of eating disorders. The focus groups and interviews were extremely rich in detail and explored many ideas in substantial depth. Focus groups included people with several different eating disorder diagnoses, including some with experience of more than 1 diagnosis, and both men and women. We also interviewed clinicians (1 man and 3 women) from a range of professional backgrounds and with varying amounts of professional experience.

Participants highlighted some concerns about the limitations of what is possible in VR, particularly around physical sensations and the sense of smell. In fact, haptics can enable participants to experience physical sensations in a VR setting [9], and it is possible to add an olfactory display to VR to allow the user to manipulate objects in VR and have an experience of smelling them [22].

### Strengths and Limitations

Although the study was small scale, we adopted a rigorous approach to data collection and analysis. Credibility was enhanced by the inclusion of 2 sample groups recruited nationally and using multiple sampling strategies to maximize variety in the perspectives obtained. Purposive sampling was used to ensure that perspectives from men were also included, thus increasing the transferability of the findings. Data analysis was carried out with triangulation between 3 researchers, each with differing backgrounds, and 1 of whom was not involved in data collection. To ensure dependability, the researchers performed independent coding and checking across both sample groups and discussed discrepancies in interpretation to arrive at a comprehensive coding schema that could be applied consistently to the data.

However, we identify the following limitations in the study. First, transferability is limited in relation to ethnicity because both our people with lived experience of eating disorders and clinician samples were limited to people who described their ethnicity as White. This was due to difficulties in recruiting people from other ethnic groups in the time available. It would be useful to expand this work to people from different ethnic groups, and with different cultural backgrounds, in the future, to open up conversations around different cultural experiences of eating disorders and VR. Second, the number of clinicians included was small, again as a result of time limitations. Their input was valuable and is novel in this type of work but it would be useful for future studies to include more clinicians. Third, it is likely that the self-selection among individuals offering to participate may have led to us recruiting participants who were more likely to be positive and enthusiastic about the potential adoption of VR. Lastly, it is possible that findings would have been different had we held interviews instead of focus groups with people with lived experience of eating disorders, and focus groups instead of interviews with clinicians. It is possible that this variation in methodologies meant we collected slightly different data from the 2 sample groups because of the format available to them in which to respond. For example, clinicians’ views may have been shaped if they had had the opportunity to interact and reflect with colleagues in situ, and people with lived experience of eating disorders may have been less able to draw on personal narratives in a group setting. Ideally, further research could triangulate these 2 methods of data collection using a mix of the 2 approaches in both sample groups. However, it is notable that the focus groups were small, and we observed individual participants talking in-depth, and also that there was a strong overlap and consistency in the content of themes obtained from the 2 sample groups.

### Comparison With Prior Work

Previous work has described a case report of a person with lived experience of eating disorders in the context of her trying VR exposure therapy in which she ate “forbidden” foods [23]. The participant described that she initially perceived the foods as too unhealthy to eat even in a virtual setting, and that she was then able to use the VR environment to practice eating feared foods [23]. However, we have been unable to find previous research which has reported qualitative research findings alongside other results in preliminary trials of VR interventions for eating disorders. We have also been unable to find other qualitative studies exploring the views of people with lived experience of eating disorders around how VR could potentially be used in treating eating disorders, despite the importance of this for ensuring that new interventions are acceptable and therefore have the potential to be effective in treating eating disorders.

There is also little research on the views of health care professionals on the potential use of VR in treating eating disorders. One survey of practicing cognitive behavioral therapists found that 45% agreed VR could be used for eating disorders (rising to 61% among those with clinical experience of treating people with eating disorders) [24]. They agreed with statements around VR enabling exposure to be tailored to the individual, increasing a sense of control, and making exposure less stressful, and shared concerns we found in our study about whether results would translate into the real world and the ability to use a new technology [24].

Early qualitative work in other areas of VR development for health-related use has found some similar general themes, particularly around the idea of VR as being novel and enjoyable [25,26]. When health care professionals are asked about potential applications for VR, they have many ideas [26].

Some qualitative work has explored the experience of people with other mental health conditions while undergoing VR interventions. These report several themes in common with our findings, such as the VR environment feeling “easier than the real thing” (p. 9) [27] or a good place to practice situations “so you cannot make a fool of yourself”(p. 4) [27]. Relatedly, they describe the potential usefulness of VR in reducing anxiety [28] and building confidence [27,28]. Caveats are also consistent, particularly the concern that it might not be for everyone, and that benefits might not translate out of a VR environment [27]. Participants were also keen to highlight that the use of VR would need to be supported by someone with whom the patient had a trusting relationship [28].

### Implications for Research and Practice

Both people with lived experience of eating disorders and clinicians in our study expressed interest and enthusiasm in the development of interventions using VR to supplement and improve the treatment of eating disorders. This work has highlighted a number of possible interventions that could be developed using VR, including psychoeducation, experiencing challenging situations, attention retraining, and seeking to make therapeutic use of the different ways VR enables one to experience one’s own body. We recommend that such interventions are co-designed with people with personal experience of eating disorders, to maximize their usefulness for this group and reduce the risk of unintended harms. Such novel interventions will subsequently require rigorous evaluation in the form of clinical trials to test their efficacy.

### Conclusions

People with personal experience of eating disorders and clinicians who are experienced in their treatment both see many potential roles for VR-based interventions in their treatment, although they are also clear that there is a need for caution and ongoing co-design in their development, particularly around how bodies are represented in a VR setting.

## Appendix

Multimedia Appendix 1 Screening questions for people with lived experience of eating disorders.

Multimedia Appendix 2 Screening questions for clinicians.

Multimedia Appendix 3 Topic guide for people with lived experience of eating disorders.

Multimedia Appendix 4 Topic guide for clinicians.

Multimedia Appendix 5 Powerpoint images shown to participants.

Multimedia Appendix 6 Practicalities of delivering virtual reality therapy for people with eating disorders.

## Abbreviations

VR: virtual reality

## Data Availability

Data are available to bona fide researchers on request via the University of Bristol Research Data Storage Facility (deposit doi: 10.5523/bris.lpf0ek7zcnxb26yzhe8a5khbx).
