# Supplementary material for: Exploring How Virtual Reality Could Be Used to Treat Eating Disorders: Qualitative Study of People With Eating Disorders and Clinicians Who Treat Them
Source: JMIR XR Spat Comput. 2024 May 14;1:e47382. doi: 10.2196/47382 (PMC12671292; doi:10.2196/47382)
Supplement: Multimedia Appendix 1 [file xr_v1i1e47382_app1.docx]

**Focus Groups on VR in Eating Disorders – Online Screening Questionnaire**

How would you describe your gender? (M/F/other (please describe))

How old are you?

How would you describe your ethnicity?

Do you have a current eating disorder? Y/N

If yes, how would you describe it (e.g. anorexia nervosa, other specified feeding and eating disorder, bulimia nervosa, binge eating disorder, etc)

If yes, how long have you had an eating disorder?

Have you previously had an eating disorder? Y/N

If yes, how would you describe it (e.g. anorexia nervosa, other specified feeding and eating disorder, bulimia nervosa, binge eating disorder, etc)

If yes, how long did your eating disorder last?

If yes, how long have you been recovered?

Are you currently having inpatient treatment in hospital for a mental or physical illness? Y/N

Are you able to speak fluent English? (we are not able to offer an interpreter)

Do you have access to a private space and an internet-enabled device (phone/tablet/computer) via which you could join a focus group?

Name:

Email:
