# Supplementary material for: Exploring How Virtual Reality Could Be Used to Treat Eating Disorders: Qualitative Study of People With Eating Disorders and Clinicians Who Treat Them
Source: JMIR XR Spat Comput. 2024 May 14;1:e47382. doi: 10.2196/47382 (PMC12671292; doi:10.2196/47382)
Supplement: Multimedia Appendix 2 [file xr_v1i1e47382_app2.docx]

**Focus Groups on VR in Eating Disorders – Online Screening Questionnaire**

How would you describe your gender? (M/F/other (please describe))

How old are you?

How would you describe your ethnicity?

Do you currently work as a health professional? Y/N

How would you describe your professional background? (e.g. mental health nurse/CPN, psychiatrist, psychologist, psychotherapist, social worker, HCA etc)

Does your current role include treating people with eating disorders? Y/N

Do you mainly work with 18+ year olds, under 18 year olds, or both?

How long have you worked with people with eating disorders?

How would you describe the main treatment interventions you use in your work with people with eating disorders? (e.g. CBT, Family Based Therapy, MANTRA, medication – please list any approaches you use)

Are you able to speak fluent English? (we are not able to offer an interpreter)

Do you have access to a private space and an internet-enabled device (phone/tablet/computer) via which you could join a focus group?

Name:

Email:
