# Supplementary material for: Exploring How Virtual Reality Could Be Used to Treat Eating Disorders: Qualitative Study of People With Eating Disorders and Clinicians Who Treat Them
Source: JMIR XR Spat Comput. 2024 May 14;1:e47382. doi: 10.2196/47382 (PMC12671292; doi:10.2196/47382)
Supplement: Multimedia Appendix 3 [file xr_v1i1e47382_app3.docx]

**Topic guide: Focus Groups on VR in Eating Disorders**

Ground rules: let everyone speak; everything remains in the group; try not to use the chat unless for a problem; put your hand up if you want to say something; we might ask individuals if they have anything to add, to give everyone a chance to speak – it’s fine to say no if you don’t. We will routinely send everyone a debrief sheet with contact details at the end of the session in case taking part raises any issues for you. If you want to leave during the session that’s fine – it would be helpful if you could send us a private message on the chat – we will follow you up with a call to check you’re ok.

Introductions (5 mins)

***LB: VR in General (15mins)***

What they understand by the term “Virtual Reality”

Any personal experiences of Virtual Reality environments

Thoughts about what a Virtual Reality environment might be like

Discussion about use of headsets – thoughts around how this would be

(Screen-sharing image of VR headset to illustrate this)

How might people be represented in a VR environment?

(Screen-sharing of VR environments)

***HB - Representing you (45mins – 1.5 hr)***

How might you want to be represented in a VR setting? (e.g. not represented at all; represented in an abstract way, e.g. maybe points of light; accurate representation of your body; representation of your body at different sizes)

Do you think how you were represented might have any effect on your eating disorder? (which aspects - your symptoms generally? your behaviour – e.g. restricting eating, binges, purging? your feelings about your body?)

***Could VR be useful/unhelpful for someone with an ED?***

What aspects might be helpful?

What aspects might be most unhelpful?

**What might you do in a VR environment** that might be helpful? (e.g. interacting with others – real or virtual – in a positive way – eg do a task or a game together/team building; interact with foods that you find hard to eat in real life; other ideas)

What might you do in a VR environment that might be unhelpful? (e.g. comparing self with others; comparing virtual “avatar” body to real body; negative interactions with others (what would this be?); other examples or thoughts).

**LB** Would you be prepared to experience being in different types of VR environment/doing different tasks in it? (10-15mins) (1.45)

What support might you need to enable you to participate in research like this?

What might you need to know to make a decision about participating?
