# Supplementary material for: Exploring How Virtual Reality Could Be Used to Treat Eating Disorders: Qualitative Study of People With Eating Disorders and Clinicians Who Treat Them
Source: JMIR XR Spat Comput. 2024 May 14;1:e47382. doi: 10.2196/47382 (PMC12671292; doi:10.2196/47382)
Supplement: Multimedia Appendix 5 [file xr_v1i1e47382_app5.pptx]

## Slide 1
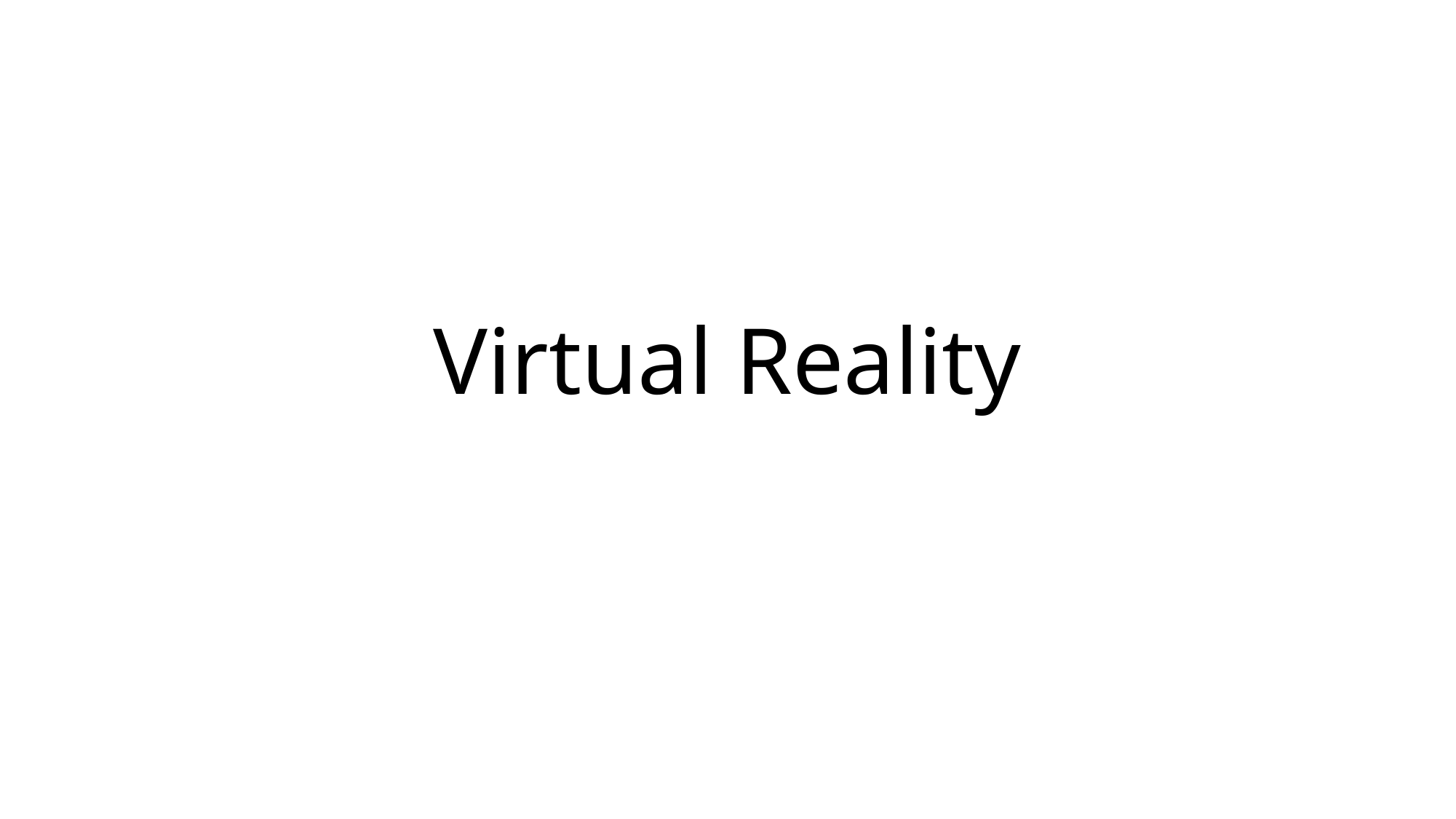

# Virtual Reality

## Slide 2
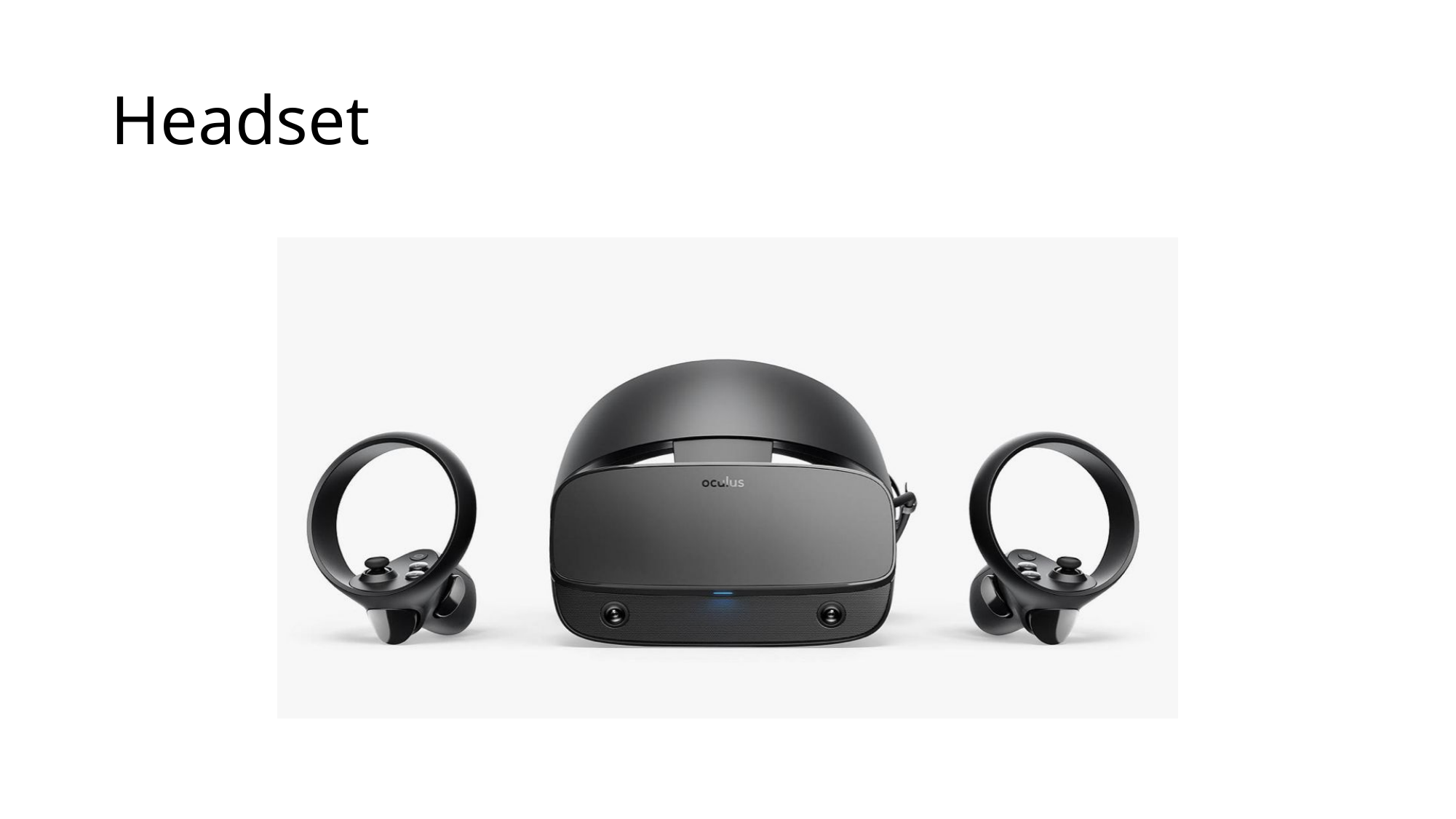

# Headset

## Slide 3
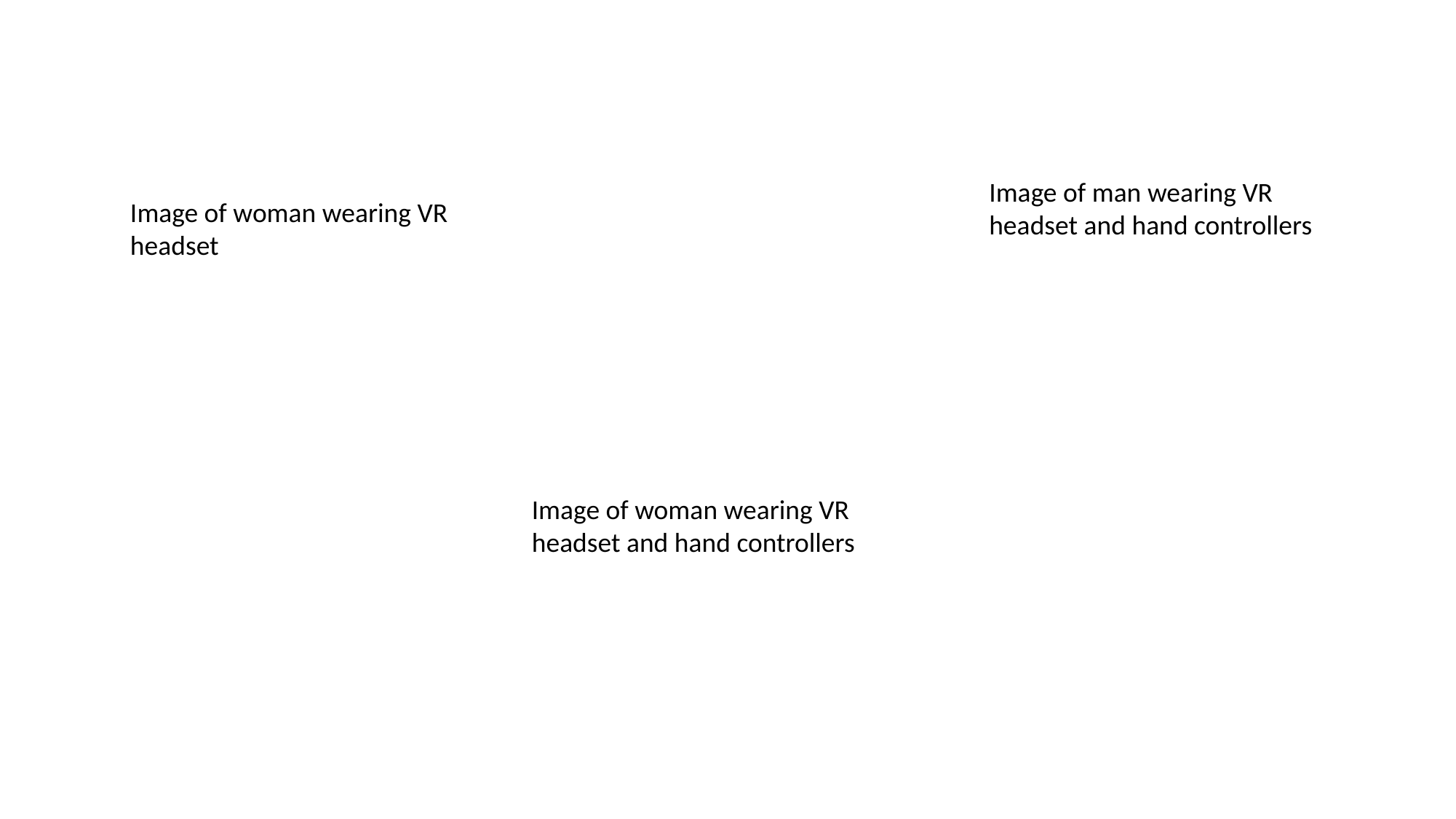

Image of man wearing VR headset and hand controllers
Image of woman wearing VR headset
Image of woman wearing VR headset and hand controllers

## Slide 4
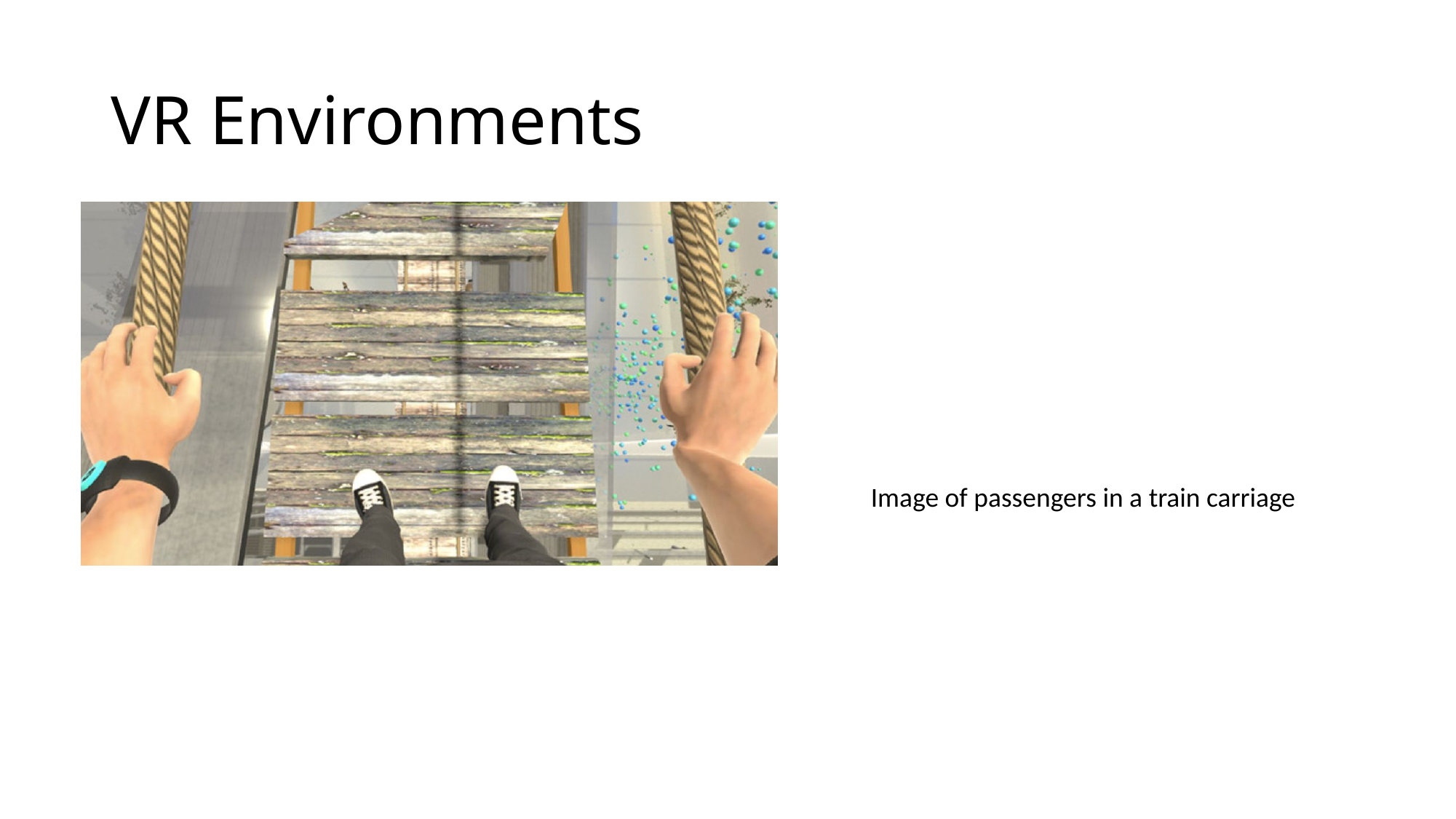

# VR Environments
Image of passengers in a train carriage

## Slide 5
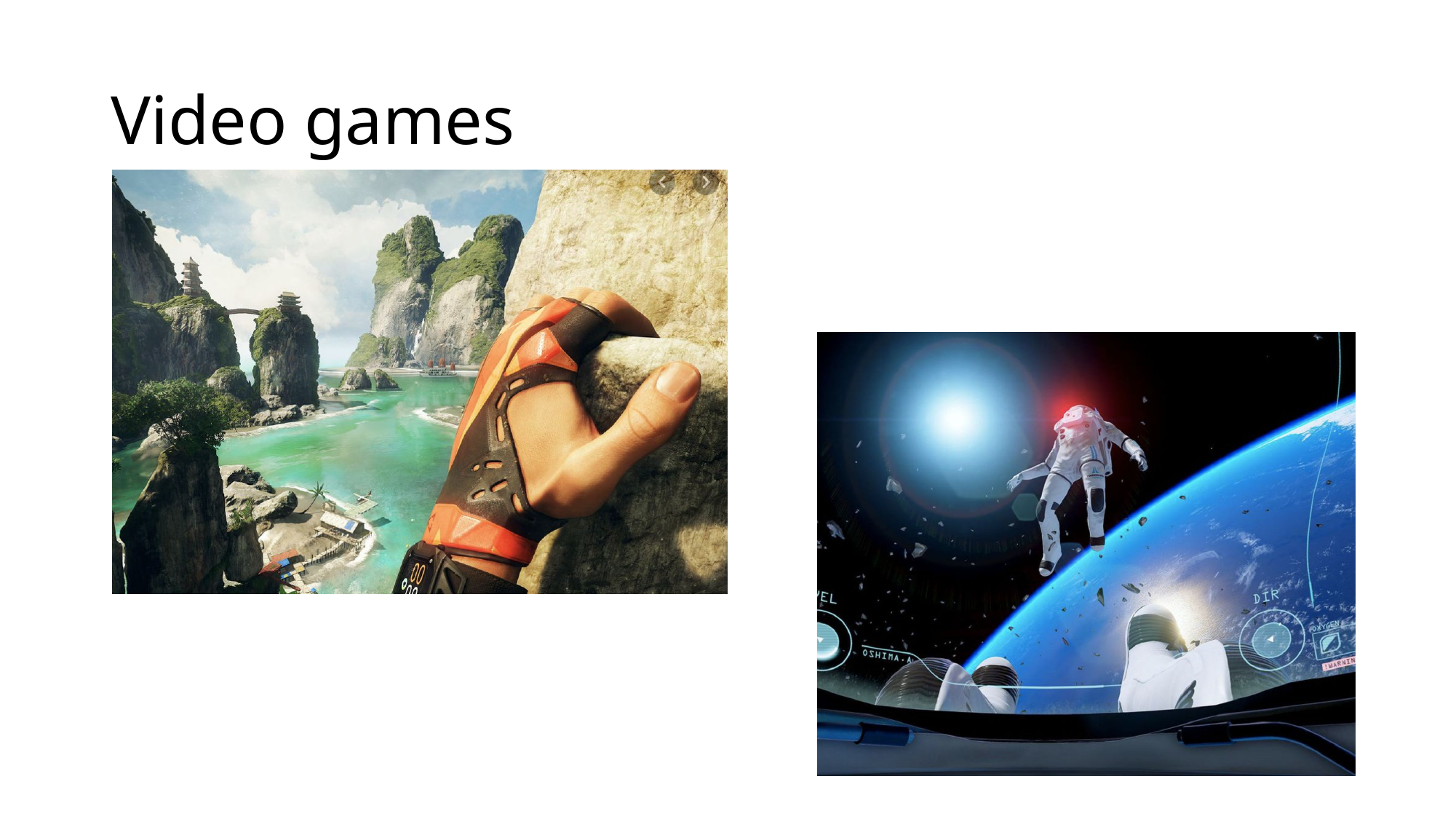

# Video games
