# Supplementary material for: Exploring How Virtual Reality Could Be Used to Treat Eating Disorders: Qualitative Study of People With Eating Disorders and Clinicians Who Treat Them
Source: JMIR XR Spat Comput. 2024 May 14;1:e47382. doi: 10.2196/47382 (PMC12671292; doi:10.2196/47382)
Supplement: Multimedia Appendix 6 [file xr_v1i1e47382_app6.docx]

**Supplementary Materials**

***Practicalities of delivering VR therapy***

*Setting*

Arguments in favour of the home environment included the idea that it might be less stressful: “*I would prefer it at home. I think I’d be much more relaxed at home*” (PWLE2); “I*’d rather try it out in my own home, so I knew sort of what it was going to be, what it was going to feel like*” (PWLE2). This was related to worries about being observed: “*if you’re on your own you haven’t got that fear of looking silly*” (PWLE1).

However, others were concerned that a distressing VR experience could intrude upon the safety associated with home: “*I don’t think I’d like the idea of that happening in my home either, cos home is kind of like a safe environment and I’d feel it could kind of cross the boundary of kind of whatever you’re exposed to in that reality and then the home environment*” (PWLE2).

One participant expressed that VR in clinic would feel out of place: “*I just feel like it’s a bit incongruous…I wouldn’t expect a therapist to give me something like this, I’d expect this to come along with like a pint of lager…you are taking something really fun and then putting it in a really formal sort of environment*” (PWLE3).

*Therapist Presence*

Many participants in both groups would want therapist involvement in or alongside any VR: “*I would always want there to be a lot of therapist involvement when I was using it… I wouldn’t ever see it as something that you can go away and do as a programme on your own*” (PWLE2); “*I can’t imagine doing it without someone being present…I imagine it would be supervised, kind of guided*” (CL0013).

This idea ran alongside concerns several PWLE expressed about trust: *“it would have to be with somebody that I trusted…I’d want my therapist to be with me on my side with it rather than like throwing me into the virtual abyss and hoping for the best”* (PWLE3); “*you’d have to trust the therapist very well and know them very well because I feel like it’s something where you’re making yourself quite vulnerable…* (PWLE2); *“I certainly wouldn’t be happy doing it in the first session with a new therapist”* (PWLE1).

Some participants in both groups suggested that whether a therapist was involved or not might depend on the particular intervention: *“with some things there are definite risks attached and probably the best way to navigate that would be to have a therapist supporting them through that”* (CL0011), and that *“I wouldn’t want to feel self-conscious doing it if they were watching but if they had an active part in it, like they were coaching you through and talking to you during it and actually being involved in it, then yes I would want someone there”* (PWLE2).

Others suggested that therapist input might be intermittent: “*Maybe there is a sort of in between…you know, they do a VR session and then, have a check in with a therapist or two VR sessions and a check in with a therapist*” (CL0011); “*I’d probably want to do stuff that I’d done in the first session…like so that I knew what I was going to do rather than like a completely new task, try this at home on your own the first time*” (PWLE3)

*Timing*

The best timing for a VR intervention was agreed to be important and complex: *“I suppose at the beginning [of treatment for someone underweight with an ED] you really want that message to be prominent, that message of food is the medicine…there can be no delay…but then…if this is kind of a way to support the people with their anxiety about it then, actually, anxiety does tend to be really high at that stage but then I guess, for children and young people the anxiety gets much higher as well when their weight is restored…that is probably a better point potentially to use something like this”* (CL0011). Some suggested it *“wouldn’t necessarily be very effective until they [patient] have restored more weight”* (CL0013); *“I think maybe when the individual’s a healthier weight…I was always told I have to be kind of a healthier weight and I think it really was valuable, you could actually kind of think better, you think more like, logically, rationally”* (PWLE2). Nutritional status may also be important: *“if someone’s not eating, I don’t think they’re often able to take things in”* (CL0013). In keeping with this, some suggested that VR would best be *used “down the line, not as a first step, I think when they’re sort of nearer recovery”* (PWLE2), or *“I see it at a time when CBT is an appropriate therapy approach…when you are ready to think about your thinking patterns and start to question those”* (PWLE2).

However, these ideas were also disputed by some *“the severity of the mental symptoms of my illness has not always correlated as you would imagine with my weight”* (PWLE1); and others suggested that VR might be helpful at any point in treatment: *“I don’t know if it would work in the community better when people are in a place where they can manage…or maybe it would always [across treatment] be relevant”* (CL0013).

Relatedly, several participants in both groups raised the patient’s current motivation for change as important: *“it’s dependent on how willing that individual is to sort of change…cause if they’re not in that right frame of mind, again, it’s just not going to be useful for them at that time”* (PWLE2) *“I think if you don’t want to change, it’s going to be a waste of time basically. You’re not going to take it seriously, you’re not going to put any effort into it”* (PWLE2). Clinicians shared this view*: “it’s so individual…someone that was coming in…and they…really did want to change the way they’re managing their eating, then yes maybe it would work”* (CL0010).
